# Arrhythmias and Hypertrophic Cardiomyopathy: Unravelling the Connection

**DOI:** 10.2174/011573403X279223231227111737

**Published:** 2024-01-24

**Authors:** Kanishk Aggarwal, Sri Pranvi Boyapati, Jayesh Valecha, Amna Noor, Fnu Kanwal, Rohit Jain, Sai Gautham Kanagala

**Affiliations:** 1 Dayanand Medical College & Hospital, Punjab, India;; 2 Siddhartha Medical College, Andhra Pradesh, India;; 3 Indira Gandhi Medical College, Shimla, India;; 4 Services Hospital Center, Lahore, Pakistan;; 5 Chandka Medical College, Larkana, Sindh, Pakistan;; 6 Penn State Milton S Hershey Medical Center, Hershey, Pennsylvania, USA;; 7 Metropolitan Hospital Center, New York, NY, USA

**Keywords:** Hypertrophic cardiomyopathy, sudden cardiac death, arrhythmias, hypertrophic cardiomyopathy (HCM), antiarrhythmic drugs, defibrillators, atrial fibrillation (AF)

## Abstract

Hypertrophic cardiomyopathy (HCM) results from gene mutations affecting cardiac sarcomeres and is inherited in an autosomal dominant manner. With a prevalence of 1:200-1:500 in the general population, HCM is characterised by a hypertrophied and non-dilated left ventricle with predominant involvement of the interventricular septum. The myocardium's structural and intracellular factors, combined with triggers such as physical exertion, autonomic dysfunction, and ischemia, can lead to reentry events, and atrial and ventricular arrhythmias, including atrial fibrillation (AF) which is common among HCM patients. To manage the increased risk of mortality arising from congestive heart failure and thromboembolism, in patients with AF long-term anticoagulation and antiarrhythmic drugs are employed. HCM patients may also encounter supraventricular and ventricular arrhythmias, such as nonsustained ventricular tachycardia and ventricular premature beats, which can potentially lead to sudden cardiac death and necessitate treatment with implanted defibrillators. Physicians must comprehensively analyse clinical, anatomical, hemodynamic, rhythmic, functional, and genetic characteristics to identify HCM patients at high risk of sudden death. This article aims to discuss the pathophysiology of arrhythmia in HCM and clinical recommendations for various ventricular and atrial fibrillation including catheter ablation and implantable cardioverter-defibrillator (ICD).

## 1 INTRODUCTION

Hypertrophic cardiomyopathy (HCM), with a prevalence of 1:200-1:500 in the general population, is a complex and relatively common inherited cardiac disease affecting millions of people worldwide without a distinct geographic or sex pattern of distribution [1, 2]. It follows an autosomal dominant pattern of inheritance and is characterised by a hypertrophied and non-dilated left ventricle with predominant involvement of the interventricular septum without evidence of any other cardiac, systemic, metabolic, or syndromic disease [3, 4]. HCM is estimated to affect around 20 million people across the world [5] and it is twice as common in black people as compared to white people in the United States [6]. In the United States alone, it is estimated that around 750,000 people may have this condition, but only about 100,000 have been diagnosed through noninvasive imaging. This suggests that many people remain undiagnosed during their lifetime, with women and underserved minorities such as blacks being particularly affected. Evidence shows that black men with undiagnosed HCM have died on the athletic field, and affected black patients may not be referred for specialized HCM-related treatments [5]. HCM is caused by more than 1400 mutations in 11 or more genes encoding proteins of the cardiac sarcomere, with MYBPC3 (Myosin Binding Protein C-3) and MYH7 (beta-Myosin Heavy Chain-7) being the most frequently mutated genes, and manifests as a spectrum of clinical features, encompassing systolic and diastolic ventricular dysfunction, cardiac arrhythmias, and sudden cardiac death, particularly in adolescents and young adults [4, 7]. The exact mechanism causing arrhythmias in HCM is unknown but has been hypothesised to result from structural and intracellular factors. Structural changes in HCM including ventricular hypertrophy, fibrosis due to microvascular ischemia, and myocyte disarray result in a chaotic or disordered myocardial architecture which disrupts the normal cardiac conduction. This along with intracellular alterations in Ca^2+^ homeostasis due to myocyte calcium hypersensitivity predispose patients with HCM to arrhythmias [8-10]. Among the arrhythmias observed in HCM patients, atrial fibrillation (AF) is the most common with patients having a four to six times higher chance of developing AF compared to the general population [3]. The incidence of AF increases with age and can reach rates as high as 40% in HCM patients over the age of 70, with an annual incidence of 2-4% and a lifetime prevalence of 20-30% [3, 11]. Additionally, HCM patients frequently experience other arrhythmias, such as supraventricular and ventricular arrhythmias, including nonsustained ventricular tachycardia and ventricular premature beats, which can lead to sudden cardiac death [12, 13]. Sudden cardiac death, with a prevalence of 0.5-1%, can even be the presenting symptom in young individuals, notably athletes [14, 15]. To mitigate the risks associated with HCM and arrhythmias, first-degree family members of HCM patients should be screened clinically with or without genetic testing. The timeline for screening is still debated within the medical community, however, it is advised to commence from the age of 12 continuing annually up to 20-25 years. For individuals aged 25 years and above, screening can be done every 3 to 5 years. This approach can be immensely useful to identify individuals requiring more intensive monitoring and treatment [1, 16]. Echocardiography remains the foundational imaging modality for HCM patients, while cardiovascular magnetic resonance imaging can be particularly useful in cases of diagnostic uncertainty or poor echocardiographic imaging windows [17]. The primary focus of treatment for obstruction in HCM is to alleviate symptoms using pharmacological or mechanical means such as septal myectomy or alcohol septal ablation. Mavacamten, a cardiac myosin ATPase inhibitor currently in Phase 3 trials, has shown promising results in improving various aspects of obstructive HCM patients. However, not all patients with LVOTO (left ventricular outflow tract obstruction) experienced the same clinical benefits, highlighting the need for a deeper understanding of the structural and functional mechanisms underlying outflow obstruction [18]. This review article aims to provide a comprehensive overview of the intricate interplay between hypertrophic cardiomyopathy and cardiac arrhythmias.

## 2 PATHOPHYSIOLOGY

Hypertrophic cardiomyopathy is associated with a heightened risk for arrhythmias, including atrial fibrillation, simple ventricular premature beats (VPB), non-sustained ventricular tachycardia (NSVT), sustained ventricular tachycardia (SVT), ventricular fibrillation (VF), hemodynamic failure and sudden cardiac death (SCD) [19]. The causes of arrhythmogenesis in hypertrophic cardiomyopathy are not entirely eluded but are thought to be a result of various factors within the structure and functioning of the myocardium. These include ventricular hypertrophy, fibrosis caused by microvascular ischemia and hypertrophy, and the disarray of myocytes, resulting in a disordered myocardial architecture. Myocyte calcium hypersensitivity may also contribute to arrhythmias, particularly when triggered by physical exertion, autonomic dysfunction, and ischemia as shown in Fig. (**1**) [9, 10].

## 3 STRUCTURAL CHANGES IN PATIENTS OF HYPERTROPHIC CARDIOMYOPATHY PREDISPOSING TO ARRHYTHMIAS

Hypertrophic cardiomyopathy, characterized by myocardial disarray and widespread myocardium fibrosis due to an increase in the rate of myocyte death and replacement with fibroblasts, often disrupts cell-to-cell alignment in the myocardium, causing delays in cardiac conduction and leading to unidirectional block, thus increasing the risk of re-entry arrhythmias like monomorphic ventricular tachycardia [20, 21]. As seen in myocardial perfusion studies, these arrhythmias are further exacerbated due to myocardial ischemia from arterial luminal thickening, leading to electrical instability in the chaotic myocyte architecture [9]. In addition, various procedures for alleviating left ventricular outflow tract obstruction (LVOTO), like alcohol septal ablation, often increase the risk of sustained ventricular arrhythmias due to myocardial scar formation [22].

## 4 INTRACELLULAR FACTORS IN PATIENTS OF HYPERTROPHIC CARDIOMYOPATHY PREDISPOSING TO ARRHYTHMIAS

For patients with HCM who have not experienced major structural changes, the risk of life-threatening ventricular arrhythmia is partially due to the malfunction of cardiomyocytes on an intracellular level [19]. Specifically, ion channel and intracellular Ca^2+^ homeostasis alterations are crucial for determining the burden of arrhythmias in HCM patients. The elevated myofilament Ca sensitivity leads to an increase in cytosolic Ca binding affinity, which can cause delayed afterdepolarizations and ventricular arrhythmias through complex mechanisms. Additionally, increased cytosolic Ca binding may alter action potential duration, like a decrease in the effective refractory period, and result in the dispersion of cardiac conduction velocities [23, 24]. These effects contribute to the development of ventricular arrhythmias. In addition, myocyte hypertrophy leads to a reduction in the inward rectifier K^+^ current (Kir2.1/IK1), transient outward K^+^ current (Kv4.3/Ito), and delayed rectifier K^+^ current (IKs, IKr) resulting in dispersion of cardiac repolarization ultimately increasing the propensity to develop arrhythmias [25, 26].

Mutations in the sarcomeres in patients with hypertrophic cardiomyopathy can cause inefficient energy utilization and a higher cost of contraction, as evidenced by cardiac magnetic resonance studies showing a 30% decrease in the phosphocreatine: ATP ratio in cardiac myocytes, indicating poor cell energy status. During exercise or other high-demand situations, this can lead to severe energy depletion and significant changes in cell electrical properties, which may explain exercise-induced arrhythmias [27].

Individuals with HCM are more susceptible to experiencing supraventricular tachycardia, specifically atrial fibrillation, as it can decrease cardiac output and afterload, resulting in symptoms of heart failure such as palpitations, shortness of breath, and chest pain. Additionally, this condition can increase the risk of thromboembolic stroke and functional deterioration, leading to higher mortality and morbidity rates. The exact pathogenesis of AF in patients with HCM is not fully understood; however, various theories have been put forward like diastolic dysfunction caused by myocyte hypertrophy and disarray, as well as reduced left ventricular compliance and increased left ventricular end-diastolic pressures, play a major role in the initiation and continuation of AF [28, 29]. Additionally, LVOTO in patients with hypertrophic obstructive cardiomyopathy, systolic anterior motion (SAM) of the mitral valve, and secondary mitral regurgitation all contribute to the enlargement of the left atrium and the occurrence of AF. The left atrial dilatation and remodeling also shorten the refractory period of the atrium and increase the dispersion of repolarization [30, 31]. Combined with genetically predisposed atrial myocyte disarray, this makes it highly susceptible to AF initiation and continuation [29]. A variant of HCM called burnt-out hypertrophic cardiomyopathy (BOHCM) occurs due to the loss of myocardium to fibrosis. It involves developing systolic dysfunction, LV dilation, and ventricular wall thinning. BOHCM is an important diagnosis to make as patients will either die or undergo heart transplants within an average of 5 years from the time this entity is identified [32].

## 5 CLINICAL APPROACH

HCM is characterized by varied clinical symptoms and phenotypic expression with varied effects on patients. While some individuals have minimal symptoms throughout their lifetime, some patients are at an increased risk of atrial and ventricular arrhythmias, which often contribute to the prognosis and mortality of the disease, with a yearly mortality rate of 1% [19, 33]. A study involving 261 patients with HCM in Japan revealed that 74 individuals (28%) had either documented paroxysmal AF (PAF) or chronic AF. AF significantly predicts mortality in HCM patients [34]. Compared to those with sinus rhythm, patients with AF have up to a fourfold increased risk of death, with most cardiovascular deaths being related to thromboembolism and worsening heart failure. In rare cases, sudden cardiac death may occur due to the deterioration of AF into ventricular tachycardia, especially in pre-excitation [34]. A study by Oliver P. *et al*. involving 4248 HCM patients found that those with AF had a higher risk of cardiovascular (10.9% AF *versus* 4.9% non-AF) and non-cardiovascular death (5.9% AF *versus* 3.2% non-AF) during a 10-year follow-up period. In addition, multiple studies have also shown that AF increases the risk of systemic thromboembolism (TE) in HCM patients [35]. In a large meta-analysis involving 7381 patients, the incidence of TE was 3.8% per year, with an overall prevalence of 27.1% [11]. In another study of 480 patients, ischemic stroke was eight times more frequent in the AF group compared to HCM patients without AF (21% AF *versus* 2.6% non-AF [34]. Individuals with HCM have a high likelihood of experiencing supraventricular and ventricular arrhythmias. A retrospective study by Amitai Segev *et al*., analysed patients with HCM and an implantable cardioverter-defibrillator (ICD) from a prospectively derived registry in two tertiary medical centers. It showed that out of 1328 patients with HCM, 207 people (145 of whom were male and had a mean age of 33 ± 16 years) were implanted with ICDs, and over a mean follow-up period of 10 ± 6 years, 18% of patients experienced sustained VTAs [36]. Sustained monomorphic VT was the most common arrhythmia, affecting 26 patients (70%), and was associated with decreased left ventricular ejection fraction and an increase in LV end-systolic and end-diastolic diameters [37] as shown in Table **1**.

Evaluation of patients with HCM often stems from positive family history, symptoms including a cardiac event, a heart murmur during physical examination, an abnormal 12-lead ECG, or incidental findings during an echocardiogram. A thorough clinical assessment includes a detailed cardiac and three-generation family history, followed by comprehensive physical examinations, incorporating maneuvers like Valsalva and squat-to-stand. An ECG and cardiac imaging follow this to confirm left ventricular hypertrophy (LVH) when clinical findings suggest HCM [17].

Screening for HCM phenotypes in family members is usually done through diagnostic imaging, starting from the age of 12 and extending up to 18-21 years. Subsequent imaging can be performed every five years unless a diagnosis is established through genetic testing. The preferred approach for screening close family relatives, especially first-degree relatives for HCM, is non-invasive imaging using an echocardiogram [1]. This method aims to identify unexplained LVH as a hallmark of the disease (phenotype) [38]. Specific patterns on a 12-lead ECG, such as ST-T abnormalities, increased voltages, deep Q waves, or pre-excitation, can indicate potential HCM even if LVH is not evident on imaging [1]. The initiation of routine echocardiographic screening for young relatives is generally advised at age 12. However, earlier screening might be necessary in cases of accelerated growth, premature puberty, family history of heart disease, symptoms suggestive of LV outflow tract obstruction, or participation in intensive sports training programs [1, 17, 39]. Implementing early identification of HCM in infants and very young children requires careful consideration due to challenges in interpreting left ventricular wall thickness relative to body size during rapid growth [17, 39, 40]. This imprecision poses a significant risk of false positive diagnoses and unnecessary recommendations, such as stopping sports activities. Therefore, 2011 ACC/AHA guidelines and the 2003 ACC/European Society of Cardiology consensus recommended that routine echocardiographic screening studies should be limited to asymptomatic family members, who are at least 12 years old, because the evidence does not support early screening, and routine screening of young children could cause unnecessary worry and expenses for otherwise healthy patients. In the context of adult family members, screening every five years is crucial to detect HCM, as it might only become apparent in young adulthood or even middle age. The severity of symptoms may vary, and it is essential to consider screening when there are abnormalities in the 12-lead ECG and genetic testing is inconclusive [1, 16, 41].

Evaluating the risk of SCD is crucial, as it is the leading cause of death among younger patients with HCM, with an annual incidence reported to be between 1-2% [42]. To effectively gauge SCD risk for adolescents and adults, a comprehensive evaluation strategy is recommended, incorporating clinical and family history, a 48-hour ambulatory ECG, Transthoracic Echocardiography (TTE), or Cardiac Magnetic Resonance (CMR) for cases with inadequate echo views, and a symptom-limited exercise test. Patients with HCM, who survive VF or sustained ventricular tachycardia, are at a high risk of subsequent lethal cardiac arrhythmias and should receive an ICD for secondary prevention. However, identifying individuals without a history of VF at increased risk of SCD remains challenging for primary prevention. To estimate the 5-year risk of SCD, it is recommended that patients undergo a standardized clinical evaluation using the HCMRisk-SCD model. The model has established three risk categories (high, intermediate, and low) based on consensus. Recommendations for ICD therapy in each category consider the statistical risk and the patient's age, general health, socio-economic factors, and psychological impact of treatment [43].

Both the European Society of Cardiology (ESC) and the American Heart Association (AHA) also advocate SCD risk stratification, during the initial assessment and periodic reassessment to track changes in SCD risk [43]. The ESC guidelines on HCM have introduced the use of a predictive score on the risk of SCD, using conventional risk markers, and include:

## 6 HISTORY, DEMOGRAPHIC, AND GENETIC RISK MARKERS

1) Age
2) Family history of SCD in one or more first-degree relatives under the age of 40 or SCD in a first-degree relative with confirmed HCM at any age.
3) History of unexplained syncope [44].

## 7 ELECTROCARDIOGRAPHIC RISK MARKERS

4) Nonsustained ventricular tachycardia (three consecutive beats at rate 120 b/min < 30 s duration on Holter monitoring).

## 8 IMAGING RISK MARKERS

5) Maximum LV wall thickness by transthoracic echocardiography.
6) Size of the left atrium by transthoracic echocardiography (LA diameter in the parasternal long axis view with M Mode or 2D).
7) Maximum LVOT gradient at rest or with provocation using pulsed and continuous wave Doppler from apical five- and three-chamber views.

Notably, the abnormal blood pressure response to exercise is no longer considered a risk factor.

Nonconventional risk markers can also be used to make decisions for ICD implantation in patients falling under the intermediate risk category and include multiple sarcomere protein gene mutations, global longitudinal strain(GLS), myocardial fibrosis ≥15% of LV mass by Late Gadolinium Enhancement (LGE) on Cardiac Magnetic Resonance (CMR), fragmented QRS complex (fQRS) on ECG, those with left ventricular apex aneurysms, transmural myocardial scarring, and thrombus formation [44-46]. Patients with a high SCD risk (>6% in 5 years) should be evaluated for ICD placement, while it is not recommended to implant ICD, if the estimated 5-year risk of SCD is less than 4%, and no other potential prognostic features are present [43].

## 9 ACC/AHA RECOMMENDATION ON ICD PLACEMENT

The ACC/AHA recommends ICD placement for secondary prevention (Class I), in select cases, including patients with HCM who have a history of cardiac arrest, ventricular fibrillation, or significant VT [1, 17]. For patients with HCM who have a high risk of sudden death, like at least one first-degree relative with HCM who suffered from sudden death, a maximum LV wall thickness of 30 mm or more, and have experienced one or more unexplained syncopal episodes, an ICD may be recommended (class IIA)[17]. ICDs can also be useful in select patients with NSVT or abnormal blood pressure response with exercise, with other SCD risk factors or modifiers, especially those under age 30. Some investigators require a minimum of two risk factors for prophylactic ICD placement [17]. Even though prophylactic ICDs are not being recommended in patients above 60 years, considering aging being the protective factor, they are advised to be screened for left ventricular apical aneurysm which is turning out to be a novel SD marker in elderly patients with HCM [47].

Before ICD implantation, patients should receive counselling on the possible dangers of inappropriate shocks, implant complications, and the social and occupational implications of an ICD. Although anti-tachycardia pacing is effective in terminating ventricular arrhythmias in HCM, it does not reduce the incidence of inappropriate shocks [17, 48, 49]. For patients with an ICD who continue to experience symptomatic ventricular arrhythmias or recurrent shocks despite optimal treatment and device reprogramming, it is recommended to use ß-blockers and amiodarone [50]. An electrophysical study is also advisable for those with ICDs and inappropriate shocks due to regular supraventricular tachycardias, to identify and treat any ablative arrhythmia substrate. Additionally, the newly developed subcutaneous ICD lead system (S-ICDTM, Boston Scientific) has FDA approval and may be considered for HCM patients with no pacing indication [43, 51]. Antiarrhythmics like amiodarone and disopyramide show limited efficacy in preventing SCD. Some patients with HCM are also advised to limit exercise due to the potential for exercise-induced ventricular arrhythmias. Avoidance of competitive sports and intense physical activity is recommended, particularly for those with SCD and LVOTO risk factors [43, 52].

## 10 MANAGEMENT OF AF IN HCM PATIENTS

Individuals with HCM and AF are also at a higher risk for thromboembolic stroke and those with AF lasting over 24 hours should receive indefinite anticoagulation [17, 43]. In comparison, those with AF episodes lasting less than 24 hours but more than 5 minutes per episode should receive anticoagulation as per current guidelines. The need for continuous anticoagulation for patients with less than 5-minute AF episodes is debatable [17]. It should be evaluated based on each patient's profile, including their bleeding risk, total AF burden, and other traditional risk factors for thromboembolism. The CHA2DS2-VASc score does not apply to HCM patients with AF since they are already at an increased risk of stroke [17, 43, 53]. Direct oral anticoagulants (DOACs) are preferred due to their effectiveness and safety over warfarin [54-56]. Pharmacotherapy and invasive therapies are used for rhythm management in HCM patients. Beta-blockers, calcium channel antagonists, and disopyramide are used for rate control, but their efficacy decreases over time [35]. Patients treated with angiotensin-converting enzyme inhibitors (ACEIs) or angiotensin receptor blockers (ARBs) appear to have a lower risk of developing new AF [57]. While ACEI/ARB can reduce the risk of developing new arrhythmias, clinical guidelines do not consider it significant, because reducing afterload can potentially worsen LVOT obstruction [58]. Catheter ablation and surgical maze procedures are used for invasive therapies in patients with symptomatic or silent episodes of AF who are unresponsive or intolerant to antiarrhythmic drug therapy [29]. Heart transplantation can be considered in patients with refractory arrhythmias or severe symptomatic heart failure. A good prognosis was observed in HCM patients who underwent heart transplants. There was an 84% survival rate observed at the year mark following the heart transplant, while survival rates at 5 years and 10 years stood at 75% and 59%, respectively [59]. In a study by Maron Ms *et al.,* comparing post-transplant survival rates between HCM patient groups and non-HCM patient subgroups observed better survival rates in HCM patients compared to non-HCM group transplanted for ischemic cardiomyopathy, but did not show much difference when compared to patients transplanted for restrictive or dilated cardiomyopathy [60, 61].

## CONCLUSION AND SUMMARY

The intricate relationship between hypertrophic cardiomyopathy (HCM) and cardiac arrhythmias holds crucial implications for future patient outcomes. Despite our evolving understanding, the exact mechanisms of arrhythmogenesis in HCM remain elusive, involving a combination of structural and intracellular factors. Atrial fibrillation (AF) emerges as a prevalent arrhythmia in HCM patients, significantly impacting morbidity and mortality. Other arrhythmias, including ventricular tachycardia and premature beats, further contribute to the disease's complexity, with sudden cardiac death being a devastating consequence. Early clinical and genetic screening of family members is vital to identify individuals at higher risk.

Arrhythmias in patients suffering from Hypertrophic cardiomyopathy (HCM) include ventricular and atria, ventricular fibrillation, ventricular tachycardia, and other supraventricular tachycardia, including atrial fibrillation. Ventricular fibrillation often poses a risk for sudden cardiac death in such patients. Implantable cardioverter-defibrillator (ICD) therapy helps prevent sudden cardiac death (SCD) without increasing mortality or worsening heart failure. The decision to implant an ICD can be challenging, but clinical judgment, informed discussions, and patients' views can help. The risk of sudden death is the same for all genders and races, but ICDs are less common in minorities. Patients with atrial fibrillation are often managed with pharmacotherapy and invasive therapies. Beta-blockers, calcium channel antagonists, and disopyramide are used for rate control. In contrast, catheter ablation and surgical maze procedures are used for invasive therapies in patients unresponsive or intolerant to antiarrhythmic drug therapy. Hypertrophic cardiomyopathy (HCM) and atrial fibrillation (AF) increase the risk of thromboembolic stroke. Those with AF episodes lasting over 24 hours should receive indefinite anticoagulation, while those with episodes lasting between 5 minutes and 24 hours should follow established guidelines. The need for continuous anticoagulation for those with less than 5 minutes of AF episodes is debatable. Heart transplantation may be an option for patients with refractory arrhythmias or severe symptomatic heart failure with a good prognosis.

## Figures and Tables

**Fig. (1):**
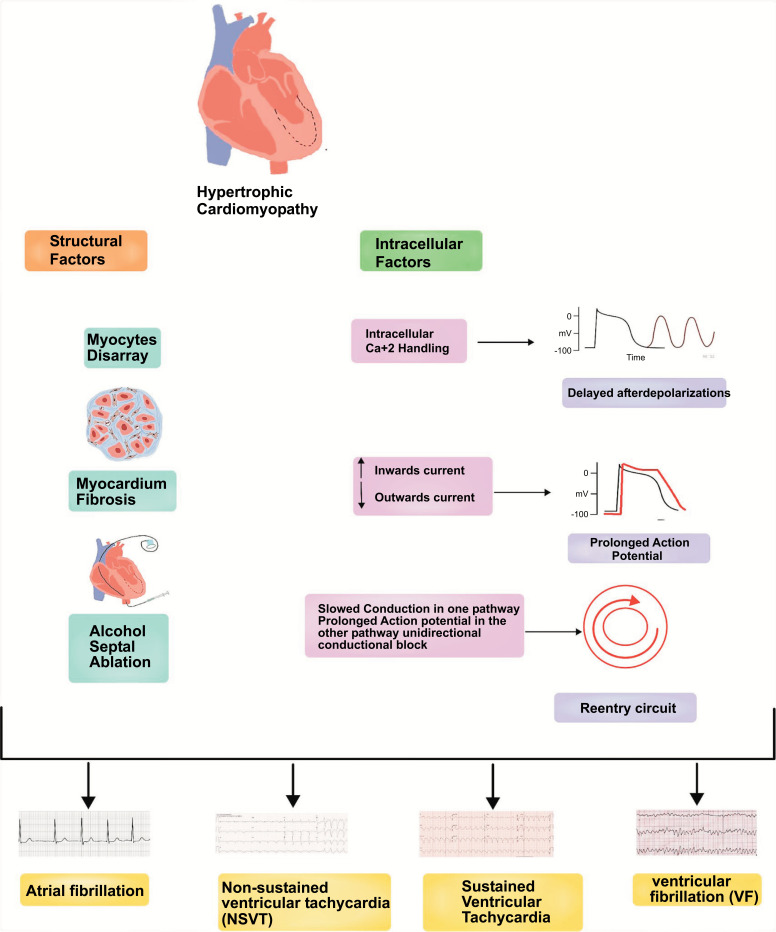
Pathophysiology of arrhythmia in HCM. **Legend:** Structural and Intracellular factors leading to various arrhythmias in Hypertrophic Cardiomyopathy.

**Table 1:** Studies illustrating the arrhythmias in patients with HCM.

**Study**	**Year Published**	**Methods**	**Conclusion**
Toru Kubo *et al.* [34]	2009	The Kochi RYOMA study, a registry for patients with cardiomyopathy, included nine hospitals where 261 patients diagnosed with HCM were registered. Of these patients, 74 (28%) had paroxysmal or chronic atrial fibrillation (AF) at registration. Most patients (93%) were classified as NYHA class I or II, but 17 of the 18 patients classified as NYHA III had AF. Among the patients with AF, 37 experienced morbid events such as embolism and heart failure (HF) admission, and 15 out of 19 patients with embolic events had AF prior to or at the time of embolism. Eight of the 29 patients with a history of HF admission had left ventricular systolic dysfunction, while the other 21 patients were admitted due to diastolic HF. Of those 21 patients, AF occurred prior to HF in 20 cases, and 19 out of those 20 patients were hospitalised within one year after the detection of AF.	In a regional registry without any specific selection criteria, it was found that AF (atrial fibrillation) was the primary factor contributing to clinical deterioration among patients diagnosed with HCM (hypertrophic cardiomyopathy).
Oliver Guttmann *et al.* [35]	2016	A retrospective, longitudinal cohort study of HCM patients collected from seven centers between 1986 and 2008 to develop multivariable Cox regression models with pre-selected predictors for Hypertrophic cardiomyopathy (HCM). Of the total patients (n=1171), 28% had coexisting hypertension. The primary objective was identifying paroxysmal, permanent, or persistent AF through ECG, Holter monitoring, or implantable device interrogation.	Of the 4248 patients with HCM without pre-existing AF, 740 (17.4%) reached the primary endpoint. Multivariable Cox regression revealed an association between AF and female sex, age, left atrial diameter, New York Heart Association (NYHA) class, hypertension, and vascular disease. The proportion of patients with cardiovascular death at ten years was 4.9% in the SR group and 10.9% in the AF group (difference in proportions = 5.9%; 95% CI (4.1% to 7.8%)). The proportion of patients with non-cardiovascular death at ten years was 3.2% in the SR group and 5.9% in the AF group (difference in proportions = 2.8%; 95% CI (0.1% to 4.2%)). An intention-to-treat propensity score analysis demonstrated that β-blockers, calcium channel antagonists, and disopyramide initially maintained SR during follow-up, but their protective effect diminished with time. Amiodarone therapy did not prevent AF during follow-up.
Oliver Guttmann *et al.* [11]	2014	Two researchers comprehensively reviewed existing literature on atrial fibrillation (AF) and thromboembolism in hypertrophic cardiomyopathy (HCM). They analyzed the prevalence and incidence of these conditions using a random effect meta-regression model and I(2) statistics. Data was independently reviewed and extracted from the identified articles	In a group of 7381 patients from 33 different studies, it was found that 22.45% (95% CI 20.13% to 24.77%) had AF. The prevalence of thromboembolism in HCM patients with AF was 27.09% (95% CI 20.94% to 33.25%). The incidence of AF was 3.08% per 100 patients per year (95% CI 2.63% to 3.54%). The incidence of thromboembolism in HCM patients with AF was 3.75% per 100 patients per year (95% CI 2.88% to 4.61%). Predictors for AF and thromboembolism included age and left atrial (LA) dimension. The LA diameter was 38.03 mm (95% CI 34.62% to 41.44%) in sinus rhythm and 45.37 mm (95% CI 41.64% to 49.04%) in AF.
Olivotto I *et al.* [36]	2001	A prospective cohort evaluating the incidence of atrial fibrillation (AF) and its impact on outcomes in a group of 480 patients with hypertrophic cardiomyopathy (HCM) with an average follow-up of 9.1+/-6.4 years.	In a study, AF was found in 107 patients (22% incidence, occurring at a rate of 2% per year). The occurrence of AF was influenced by increasing age, congestive symptoms, and larger LA size at the time of diagnosis. Patients with AF had a higher risk of HCM-related death (OR 3.7, *P*<0.002) due to an increased risk of heart failure-related mortality, but not sudden or unexpected death. The risk associated with AF was even more significant in patients with outflow obstruction or who developed AF at or before 50. AF patients were also at increased risk for stroke (OR 17.7, *P*=0.0001) and severe functional limitation (OR for NYHA class III or IV, 2.8, *P*<0.0001). Patients with chronic AF had a higher probability of HCM-related death, functional impairment, and stroke than those with exclusively paroxysmal AF (*P*<0.0001). However, 37 patients with AF (35%) had a relatively benign clinical course without stroke and severe symptoms.
Amitai Segev *et al.* [37]	2014	A retrospective analysis of patients with HCM who had an implantable cardioverter-defibrillator (ICD) from a registry at two tertiary medical centers was conducted and collected and compared clinical, electrocardiographic, echocardiographic, ICD interrogation, and genetic data of patients with and without VTAs and then between patients with only VF and those with ventricular tachycardia (VT) with or without VF.	Out of the 1,328 individuals with HCM, 207 (145 of which were male, making up 70% of the group, with an average age of 33 ± 16 years) received ICD implants. During an average follow-up period of 10 ± 6 years, 37 patients (18%) with ICDs developed sustained VTAs. A family history of sudden cardiac death and a personal history of VTAs were linked to this occurrence (with P values of 0.036 and 0.001, respectively). Sustained monomorphic VT was the most common type of arrhythmia affecting 26 individuals (70%) and was connected to decreased left ventricular ejection fraction and increased left ventricular end-systolic and end-diastolic diameters. Antitachycardia pacing (ATP) successfully terminated 258 (79%) of the 326 VT events. Mortality rates were similar between individuals with and without VTAs (4 [11%] *versus* 29 [17%], respectively; *P* = 0.42) and between those with and without ICDs (24 [16%] *versus* 85 [20%]; P = 0.367)

**Note:** Studies showing the correlation between HCM and cardiac arrhythmias.

## LIST OF ABBREVIATIONS

AF: Atrial Fibrillation
HCM: Hypertrophic Cardiomyopathy
ICD: Implantable Cardioverter-Defibrillator
TE: Thromboembolism

## References

[1] Maron B.J., Desai M.Y., Nishimura R.A. (2022). Diagnosis and evaluation of hypertrophic cardiomyopathy.. J. Am. Coll. Cardiol..

[2] Marian A.J., Braunwald E. (2017). Hypertrophic cardiomyopathy.. Circ. Res..

[3] Garg L., Gupta M., Sabzwari S.R.A. (2019). Atrial fibrillation in hypertrophic cardiomyopathy: Prevalence, clinical impact, and management.. Heart Fail. Rev..

[4] Maron B.J., Maron M.S. (2013). Hypertrophic cardiomyopathy.. Lancet.

[5] Maron B.J. (2018). Clinical course and management of hypertrophic cardiomyopathy.. N. Engl. J. Med..

[6] Ntusi N.A.B., Sliwa K. (2021). Associations of race and ethnicity with presentation and outcomes of hypertrophic cardiomyopathy.. J. Am. Coll. Cardiol..

[7] Gartzonikas I.K., Naka K.K., Anastasakis A. (2022). Current and emerging perspectives on pathophysiology, diagnosis, and management of hypertrophic cardiomyopathy.. Hellenic J. Cardiol..

[8] Bahrudin U., Morikawa K., Takeuchi A. (2011). Impairment of ubiquitin-proteasome system by E334K cMyBPC modifies channel proteins, leading to electrophysiological dysfunction.. J. Mol. Biol..

[9] Basso C., Thiene G., Corrado D., Buja G., Melacini P., Nava A. (2000). Hypertrophic cardiomyopathy and sudden death in the young: Pathologic evidence of myocardial ischemia.. Hum. Pathol..

[10] Kibos A.S., Knight B.P., Vidal Essebag, Fishberger SB., Slevin M. (2014). Țintoiu IC cardiac arrhythmias from basic mechanism to state-of-the-art management..

[11] Guttmann O.P., Rahman M.S., O’Mahony C., Anastasakis A., Elliott P.M. (2014). Atrial fibrillation and thromboembolism in patients with hypertrophic cardiomyopathy: Systematic review.. Heart.

[12] Kuck K.H. (1997). Arrhythmias in hypertrophic cardiomyopathy.. Pacing Clin. Electrophysiol..

[13] Kumar K.R., Mandleywala S.N., Link M.S. (2015). Atrial and ventricular arrhythmias in hypertrophic cardiomyopathy.. Card. Electrophysiol. Clin..

[14] Moore B., Semsarian C., Chan K.H., Sy R.W. (2019). Sudden cardiac death and ventricular arrhythmias in hypertrophic cardiomyopathy.. Heart Lung Circ..

[15] Shirani J., Pick R., Roberts W.C., Maron B.J. (2000). Morphology and significance of the left ventricular collagen network in young patients with hypertrophic cardiomyopathy and sudden cardiac death.. J. Am. Coll. Cardiol..

[16] Gersh B.J., Maron B.J., Bonow R.O. (2011). 2011 ACCF/AHA guideline for the diagnosis and treatment of hypertrophic cardiomyopathy: executive summary: A report of the american college of cardiology foundation/american heart association task force on practice guidelines.. Circulation.

[17] Ommen S.R., Mital S., Burke M.A. (2020). AHA/ACC Guideline for the diagnosis and treatment of patients with hypertrophic cardiomyopathy.. Circulation.

[18] Hermida U., Stojanovski D., Raman B. (2023). Left ventricular anatomy in obstructive hypertrophic cardiomyopathy: Beyond basal septal hypertrophy.. Eur. Heart J. Cardiovasc. Imaging.

[19] Shen H., Dong S.Y., Ren M.S., Wang R. (2022). Ventricular arrhythmia and sudden cardiac death in hypertrophic cardiomyopathy: From bench to bedside.. Front. Cardiovasc. Med..

[20] Elliott P., Spirito P. (2007). Prevention of hypertrophic cardiomyopathy-related deaths: Theory and practice.. Heart.

[21] Michels M. (2012). Appropriate implantable cardioverter defibrillator therapy in hypertrophic cardiomyopathy: What happens on Sunday afternoons in May?. Europace.

[22] Bockstall K.E., Link M.S. (2012). A primer on arrhythmias in patients with hypertrophic cardiomyopathy.. Curr. Cardiol. Rep..

[23] Baudenbacher F., Schober T., Pinto J.R. (2008). Myofilament Ca2+ sensitization causes susceptibility to cardiac arrhythmia in mice.. J. Clin. Invest..

[24] Schober T., Huke S., Venkataraman R. (2012). Myofilament Ca sensitization increases cytosolic Ca binding affinity, alters intracellular Ca homeostasis, and causes pause-dependent Ca-triggered arrhythmia.. Circ. Res..

[25] Coppini R., Ferrantini C., Yao L. (2013). Late sodium current inhibition reverses electromechanical dysfunction in human hypertrophic cardiomyopathy.. Circulation.

[26] Yang K.C., Jay P.Y., McMullen J.R., Nerbonne J.M. (2012). Enhanced cardiac PI3Kα signalling mitigates arrhythmogenic electrical remodelling in pathological hypertrophy and heart failure.. Cardiovasc. Res..

[27] Frey N., Luedde M., Katus H.A. (2012). Mechanisms of disease: Hypertrophic cardiomyopathy.. Nat. Rev. Cardiol..

[28] Ali A., Pecha S., Aydin A. (2018). Atrial fibrillation in hypertrophic cardiomyopathy: diagnosis and considerations for management.. J. Atr. Fibrillation.

[29] Dragasis S., Vlachos K., Kariki O. (2022). Atrial fibrillation in hypertrophic cardiomyopathy - A contemporary mini-review. Hellenic journal of cardiology: HJC =. Hellenike kardiologike epitheorese..

[30] Prinz C., Vanbuuren F., Bogunovic N., Bitter T., Faber L., Horstkotte D. (2012). In patients with hypertrophic cardiomyopathy myocardial fi brosis is associated with both left ventricular and left atrial dysfunction.. Acta Cardiol..

[31] Papavassiliu T., Germans T., Flüchter S. (2009). CMR findings in patients with hypertrophic cardiomyopathy and atrial fibrillation.. J. Cardiovasc. Magn. Reson..

[32] Rahmani G., Kraushaar G., Dehghani P. (2015). Diagnosing burned-out hypertrophic cardiomyopathy: Daughter’s phenotype solidifies father’s diagnosis.. J. Cardiol. Cases.

[33] Mielczarek S., Syska P., Lewandowski M., Kowalik I., Pytkowski M., Szwed H. (2022). Long-term mortality and risk factor analysis in hypertrophic cardiomyopathy patients with implantable cardioverter-defibrillators.. Polish Archives of Internal Medicine.

[34] Kubo T., Kitaoka H., Okawa M. (2009). Clinical impact of atrial fibrillation in patients with hypertrophic cardiomyopathy. Results from Kochi RYOMA Study.. Circ. J..

[35] Guttmann O.P., Pavlou M., O’Mahony C. (2017). Predictors of atrial fibrillation in hypertrophic cardiomyopathy.. Heart.

[36] Olivotto I., Cecchi F., Casey S.A., Dolara A., Traverse J.H., Maron B.J. (2001). Impact of atrial fibrillation on the clinical course of hypertrophic cardiomyopathy.. Circulation.

[37] Segev A., Wasserstrum Y., Arad M. (2023). Ventricular arrhythmias in patients with hypertrophic cardiomyopathy: Prevalence, distribution, predictors, and outcome.. Heart Rhythm.

[38] Rowin E.J., Maron B.J., Maron M.S. (2020). The hypertrophic cardiomyopathy phenotype viewed through the prism of multimodality imaging.. JACC Cardiovasc. Imaging.

[39] Maron B.J., Seidman J.G., Seidman C.E. (2004). Proposal for contemporary screening strategies in families with hypertrophic cardiomyopathy.. J. Am. Coll. Cardiol..

[40] Norrish G., Jager J., Field E. (2019). Yield of clinical screening for hypertrophic cardiomyopathy in child first-degree relatives.. Circulation.

[41] Maron B.J., McKenna W.J., Danielson G.K. (2003). American college of cardiology/european society of cardiology clinical expert consensus document on hypertrophic cardiomyopathy.. J. Am. Coll. Cardiol..

[42] Elliott P.M., Gimeno J.R., Thaman R. (2005). Historical trends in reported survival rates in patients with hypertrophic cardiomyopathy.. Heart.

[43] Elliott P.M., Anastasakis A., Borger M.A. (2014). 2014 ESC Guidelines on diagnosis and management of hypertrophic cardiomyopathy.. Eur. Heart J..

[44] Makavos G., Κairis C., Tselegkidi M.E. (2019). Hypertrophic cardiomyopathy: An updated review on diagnosis, prognosis, and treatment.. Heart Fail. Rev..

[45] Chan R.H., Maron B.J., Olivotto I. (2014). Prognostic value of quantitative contrast-enhanced cardiovascular magnetic resonance for the evaluation of sudden death risk in patients with hypertrophic cardiomyopathy.. Circulation.

[46] Maron M.S., Finley J.J., Bos J.M. (2008). Prevalence, clinical significance, and natural history of left ventricular apical aneurysms in hypertrophic cardiomyopathy.. Circulation.

[47] Rowin E.J., Maron B.J., Chokshi A., Maron M.S. (2018). Left ventricular apical aneurysm in hypertrophic cardiomyopathy as a risk factor for sudden death at any age.. Pacing Clin. Electrophysiol..

[48] Roberts B.D., Hood R., Saba M.M., Dickfeld T.M., Saliaris A.P., Shorofsky S.R. (2010). Defibrillation threshold testing in patients with hypertrophic cardiomyopathy.. Pacing Clin. Electrophysiol..

[49] Nagai T., Kurita T., Satomi K. (2009). QRS prolongation is associated with high defibrillation thresholds during cardioverter-defibrillator implantations in patients with hypertrophic cardiomyopathy.. Circ. J..

[50] Zipes D.P., Camm A.J., Borggrefe M. (2006). ACC/AHA/ESC 2006 guidelines for management of patients with ventricular arrhythmias and the prevention of sudden cardiac death.. J. Am. Coll. Cardiol..

[51] Bardy G.H., Smith W.M., Hood M.A. (2010). An entirely subcutaneous implantable cardioverter-defibrillator.. N. Engl. J. Med..

[52] Gimeno J.R., Tomé-Esteban M., Lofiego C. (2009). Exercise-induced ventricular arrhythmias and risk of sudden cardiac death in patients with hypertrophic cardiomyopathy.. Eur. Heart J..

[53] Nazer B., Dale Z., Carrassa G. (2020). Appropriate and inappropriate shocks in hypertrophic cardiomyopathy patients with subcutaneous implantable cardioverter-defibrillators: An international multicenter study.. Heart Rhythm.

[54] Patel N., Elzanaty A., Patel M., Elsheikh E. (2021). Efficacy and safety of direct anticoagulants *versus* vitamin K antagonists in patients with atrial fibrillation and bioprosthetic valves: A systematic review and meta-analysis.. J. Am. Coll. Cardiol..

[55] Nasser M.F., Gandhi S., Siegel R.J., Rader F. (2020). Anticoagulation for stroke prevention in patients with hypertrophic cardiomyopathy and atrial fibrillation: A review.. Heart Rhythm.

[56] Lin Y., Xiong H., Su J. (2022). Effectiveness and safety of non-vitamin K antagonist oral anticoagulants in patients with hypertrophic cardiomyopathy with non-valvular atrial fibrillation.. Heart Vessels.

[57] Huang C.Y., Yang Y.H., Lin L.Y. (2018). Renin–angiotensin–aldosterone blockade reduces atrial fibrillation in hypertrophic cardiomyopathy.. Heart.

[58] Olivotto I., Cecchi F., Poggesi C., Yacoub M.H. (2012). Patterns of disease progression in hypertrophic cardiomyopathy: An individualized approach to clinical staging.. Circ. Heart Fail..

[59] Torres M.F., Perez-Villa F. (2018). Heart transplantation in patients with hypertrophic cardiomyopathy.. Glob. Cardiol. Sci. Pract..

[60] Kato T.S., Takayama H., Yoshizawa S. (2012). Cardiac transplantation in patients with hypertrophic cardiomyopathy.. Am. J. Cardiol..

[61] Maron M.S., Kalsmith B.M., Udelson J.E., Li W., DeNofrio D. (2010). Survival after cardiac transplantation in patients with hypertrophic cardiomyopathy.. Circ. Heart Fail..

